# Loss of chromosome 11q21–23.1 and 17p and gain of chromosome 6p are independent prognostic indicators in B-cell non-Hodgkin's lymphoma

**DOI:** 10.1054/bjoc.2001.2164

**Published:** 2001-12

**Authors:** T Stokke, P DeAngelis, L Smedshammer, E Galteland, H B Steen, E B Smeland, J Delabie, H Holte

**Affiliations:** Departments of Biophysics, Immunology, Pathology, Oncology, The Norwegian Radium Hospital, Oslo, 0310, Norway; Department of Pathology, The Norwegian National Hospital, Oslo, 0027, Norway

**Keywords:** non-Hodgkin's lymphoma, chromosomal aberrations, survival

## Abstract

Comparative genomic hybridization (CGH) was employed to study chromosomal aberrations in relation to cell proliferation, apoptosis, and patient survival in 94 cases of B-cell non-Hodgkin's lymphoma diagnosed between 1983 and 1993. Eighty cases had aberrations by CGH. Chromosomal regions 1p21–31.1 (10%), 6cen-q24 (12%), 8p (11%), 9p21-ter (14%), 11q21–23.1 (11%), 13q13–21.1 (12%), and 17p (15%) were frequently lost. Gains were found at 3q21-ter (22%), 6p (11%), 7p (12%), 8q23-ter (13%), 12cen-q15 (17%), 17q24-ter (13%), and 18q13.3–21 (20%). A high number of aberrations (≥ 4, 33 cases) was associated (*P* ≤ 0.001) with the mantle cell and diffuse large B-cell lymphoma subtypes, a high fraction of tumour cells in S phase, and short survival (RR (relative risk) = 3.7). Loss of 1p21–31.1, 8p, 9p21-ter, 11q21–23.1, and 13q13–21.1 were associated with mantle cell lymphoma (*P* ≤ 0.03), while gain of 6p and 12cen-q15 were more frequent in diffuse large B-cell and small lymphocytic lymphoma, respectively (*P* = 0.04). Loss of 8p and 17p, and gain of 3q21-ter, 6p, 7p, and 8q23-ter were associated with a high S phase fraction (*P* ≤ 0.03), but none of the aberrations were associated with tumour apoptotic fraction (*P* ≥ 0.13). The most important prognostic CGH parameters (*P* < 0.001) were losses of 11q21–23.1 (RR = 3.8) and 17p (RR = 4.4), and gain of 6p (RR = 4.2). The latter parameters and IPI were the only ones with independent prognostic value (RR = 10, 5.0, 6.7, and 3.7, respectively; *P* < 0.001) when assessed together with lymphoma sub-type, primary versus relapse cases, treatment, B symptoms, S phase fraction, and presence of BCL1 and BCL2 translocations. A combined CGH/IPI binary parameter had high prognostic value for patients receiving different treatments, with various lymphoma sub-types, and for primary as well as relapse cases.© 2001 Cancer Research Campaign http://www.bjcancer.com


*British Journal of Cancer* (2001) **85**(12), 1900–1913
© 2001 Cancer Research Campaign
doi: 10.1054/ bjoc.2001.2164, available online at http://www.idealibrary.com on IDEAL

http://www.bjcancer.com

### Loss of chromosome 11q21–23.1 and 17p and gain of chromosome 6p are independent prognostic indicators in B-cell non-Hodgkin's lymphoma

**T Stokke[1], P DeAngelis[2], L Smedshammer[1], E Galteland[1], HB Steen[1], EB Smeland[3], J Delabie[4], and H Holte[5]**

Departments of [1]Biophysics, [3]Immunology, [4]Pathology and [5]Oncology, The Norwegian Radium Hospital, 0310 Oslo, Norway; and [2]Department of Pathology, The Norwegian National Hospital, 0027 Oslo, Norway

**Summary** Comparative genomic hybridization (CGH) was employed to study chromosomal aberrations in relation to cell proliferation, apoptosis, and patient survival in 94 cases of B-cell non-Hodgkin's lymphoma diagnosed between 1983 and 1993. Eighty cases had aberrations by CGH. Chromosomal regions 1p21–31.1 (10%), 6cen-q24 (12%), 8p (11%), 9p21-ter (14%), 11q21–23.1 (11%), 13q13–21.1 (12%), and 17p (15%) were frequently lost. Gains were found at 3q21-ter (22%), 6p (11%), 7p (12%), 8q23-ter (13%), 12cen-q15 (17%), 17q24-ter (13%), and 18q13.3–21 (20%). A high number of aberrations (≥ 4, 33 cases) was associated ($P \leq 0.001$) with the mantle cell and diffuse large B-cell lymphoma subtypes, a high fraction of tumour cells in S phase, and short survival (RR (relative risk) = 3.7). Loss of 1p21–31.1, 8p, 9p21-ter, 11q21–23.1, and 13q13–21.1 were associated with mantle cell lymphoma ($P \leq 0.03$), while gain of 6p and 12cen-q15 were more frequent in diffuse large B-cell and small lymphocytic lymphoma, respectively ($P = 0.04$). Loss of 8p and 17p, and gain of 3q21-ter, 6p, 7p, and 8q23-ter were associated with a high S phase fraction ($P \leq 0.03$), but none of the aberrations were associated with tumour apoptotic fraction ($P \geq 0.13$). The most important prognostic CGH parameters ($P < 0.001$) were losses of 11q21–23.1 (RR = 3.8) and 17p (RR = 4.4), and gain of 6p (RR = 4.2). The latter parameters and IPI were the only ones with independent prognostic value (RR = 10, 5.0, 6.7, and 3.7, respectively; $P < 0.001$) when assessed together with lymphoma sub-type, primary versus relapse cases, treatment, B symptoms, S phase fraction, and presence of BCL1 and BCL2 translocations. A combined CGH/IPI binary parameter had high prognostic value for patients receiving different treatments, with various lymphoma sub-types, and for primary as well as relapse cases. © 2001 Cancer Research Campaign http://www.bjcancer.com

**Keywords**: non-Hodgkin's lymphoma; chromosomal aberrations, survival

Non-Hodgkin's lymphoma (NHL) is clinically a heterogeneous group of malignancies for which the B-cell phenotype constitutes 85% of the cases. For the indolent non-localized lymphomas there is no curative treatment, but median overall survival is close to 10 years and differs markedly between patients. Approximately 40% of aggressive NHL are cured by combination chemotherapy (Fisher et al, 1993). Lymphoma sub-types (Harris et al, 1994) and prognostic factors like stage, age, serum LDH, WHO performance, and number of extranodal sites (The International Non-Hodgkin's Lymphoma Prognostic Factors Project, 1993) are presently the tools for treatment selection and the background for informing the patient on life expectancy.

The development of B-cell NHL is, as for other neoplasias, accompanied by the sequential acquisition of monoclonal genetic aberrations. Typical aberrations in B-cell NHL include translocations involving the immunoglobulin loci and other chromosomal loci harbouring proto-oncogenes like c-MYC, BCL-1, BCL-2, and BCL-6 (Rabbitts, 1994; Ong and Le Beau, 1998). These translocations activate inappropriate transcription of the proto-oncogene involved, and may, together with loss of tumour suppressor activities, lead to deregulated growth due to increased proliferation or decreased apoptosis. Losses and gains of tumour suppressor genes and proto-oncogenes or larger genomic regions containing such genes are common events in the genesis of most solid tumours. Classical cytogenetic studies have revealed that unbalanced translocations and gains and losses of whole chromosomes are frequent also in NHL (Offit, 1992; Mitelman et al, 1997; Ong and Le Beau, 1998).

Comparative genomic hybridization (CGH) is based on hybridization of tumour DNA from interphase cells to normal metaphases for the detection of gain or loss of specific chromosomal regions. CGH does not provide any information about the chromosomal organization of the tumour genome, but has several advantages compared to classical cytogenetics: Interphase cells can be analysed without the need for culturing to make metaphase spreads; The composition of complex marker chromosomes, which can be difficult to analyse by cytogenetics, is easily obtained by CGH. In agreement with the classical cytogenetic studies, CGH studies of B-cell NHL have shown genomic losses and gains to be frequent in follicle centre lymphomas (FCL, Bentz et al, 1996), small lymphocytic lymphomas/chronic lymphocytic leukemias (SLL/CLL, Bentz et al, 1995), mantle cell lymphomas (MCL, Monni et al, 1998; Beà et al, 1999; Bentz et al, 2000), and diffuse large B cell lymphomas (DLBCL, Monni et al, 1996).

Specific sub-types of NHL are characterized by specific chromosomal translocations. However, it is less well known whether additional chromosomal changes are also confined to certain lymphoma sub-types, and whether these correlate with prognosis. The results of numerous reports strongly suggest that the presence

*Received 11 September 2001*
*Revised 11 September 2001*
*Accepted 20 September 2001*

*Correspondence to:* T Stokke of aberrations in the p53 pathway (i.e. 17p deletions, TP53 mutations, expression of wild-type p53) are adverse prognostic factors in NHL (Sander et al, 1993; Tilly et al, 1994; Dohner et al, 1995; Schoch et al, 1995; Hernandez et al, 1996; Panayiotidis and Kotsi, 1999; Dohner et al, 2000; Stokke et al, 2000; reviewed by Knutsen, 1998). Chromosomal aberrations are important prognostic factors and are used for treatment stratification in acute lymphoblastic leukaemia, including the indication for bone marrow transplantation in first remission (Pui and Evans, 1998).

We report here the findings of a CGH study of 94 cases of randomly selected B-cell NHL. The chromosomal gains and losses were compared with lymphoma subtype, S phase and apoptotic fraction, as well as prognosis.

### MATERIALS AND METHODS

### Patients

The 94 patients included in this study were randomly selected based on the diagnosis of B-cell non-Hodgkin's lymphoma and on the availability of frozen cell suspensions (in DMSO) with more than 50% tumour cells (Ig-expressing cells by manual counting and flow cytometry). All patients were hospitalized at our institution during the period 1983–1993. Lymphoma subtyping was performed according to the REAL classification (Harris et al, 1994). Twelve of the 13 MCL cases in this study had cyclin D1 expression and t(11;14) (lacking in case 67/92), and 32 of the 36 FCL cases had t(14;18) (lacking in cases 577/90, 489/91, 533/91, and 300/92).

Clinical data were available for 92 of the patients (lacking in cases 159/90 and 244/91). The age range of these patients at the time of the relevant biopsy was 31–86 years, the median age was 57 years, and 41 of the patients were women (42 of the total of 94). For 62 patients, the biopsy was taken at diagnosis, for 15 patients at first relapse, and for 15 patients at second or later relapse/progression. No patients had received chemotherapy during the last month before the biopsies. The median follow-up of the 31 survivors was 59 months. Clinical stage was according to the Ann Arbor classification (Carbone et al, 1971) at the time of diagnosis. The patients were clinically examined and received standard blood testing, standard X-ray of the thorax, CT scan of the abdomen/pelvis and, if necessary, of the thorax, as well as bilateral bone marrow aspiration/biopsy.

Patients with DLBCL and BL were treated according to the following protocols: Eight courses of CHOP (cyclophosphamide 750 mg/m$^2$ i.v. day 1, doxorubicin 50 mg/m$^2$ i.v. day 1, vincristin 2 mg day 1, and prednisone 100 mg p.o. day 1–5) for stage III–IV, and 6 courses of CHOP followed by involved field radiotherapy for patients with stage I disease. If two neighbouring lymph node regions were involved, stage II DLBCL and BL patients were treated as stage I patients, otherwise, treatment was as for stage III–IV. For the SLL, MZL, FCL, and MCL lymphomas, the following treatment was given: When required, initial therapy to the patients consisted of radiotherapy to stage I–II disease, while patients with stage III–IV disease were given the CVP regimen (cyclophosphamide 750 mg/m$^2$ i.v. day 1, vincristin 2 mg day 1, and prednisone 100 mg p.o. day 1–5) during the early part of the study period, whereas chlorambucil 6 mg/m$^2$ p.o. and prednisone 5–10 mg p.o. daily for up to 6 months was given during the latter part of the period. Younger patients with an aggressive clinical course were given CHOP at the discretion of the responsible clinician. The CVP and CHOP regimens were repeated every 3 weeks for a total of 6–8 courses; the chemotherapy regimen was changed if at least a partial remission was not achieved within 2–3 months of therapy. Treatment at relapse or progression was given at the discretion of the responsible clinician. No patients had received chemotherapy during the last month before the biopsies were taken.

### Comparative genomic hybridization

The isolation of genomic DNA from the (thawed) lymphoma samples was performed after a standard protocol based upon proteinase K and dodecyl sulphate as described by DeAngelis et al (1999). The DNA concentration was determined by Hoechst 33258 staining and measuring the fluorescence in a fluorimeter.

Genomic DNA was nick-translated and labelled with either Texas red-5-dUTP for normal sex-matched DNA or FITC-12-dUTP for tumor DNA (Kallioniemi et al, 1994a; DeAngelis et al, 1999).

The slides for CGH hybridization were prepared following routine procedures for PHA stimulated, methotrexate synchronized, peripheral blood lymphocytes. After dripping the cells onto the slides at 60–65% relative humidity, the slides were stored at –20˚C in 100% ethanol or at 0˚C in a nitrogen flushed container.

Denaturation, hybridization, and counterstaining with DAPI was essentially performed as outlined by Kallioniemi et al (1994a), and in more detail as described by DeAngelis et al (1999).

### Microscopy and data treatment

DAPI fluorescence and probe signals were observed sequentially with a Zeiss Axioplan fluorescence microscope equipped with a triple-pass emission filter (blue, green and red), a corresponding beam splitter, and separate excitation filters (UV, 470–490 nm, 578 nm). All filters ('Pinkel 1' filter set) were obtained from Chroma, Brattleboro, VT. Images were captured and digitized in a cooled 16 bit CCD camera (Astromed, Cambridge, UK).

Segmentation and calculation of ratio profiles were performed with software that was kindly provided by D. Sudar. The software ran under the 'Scilimage' image analysis program (TNO, Delft, Netherlands). Hybridizations were repeated when the FITC or the Texas Red chromosomal fluorescence was low (signal to background less than ~ 2), or grainy, or when blocking of (labelled) probe hybridization to centromere regions and the p-arms of acrocentrics was not satisfactory. Each tumour was satisfactory. Each tumour was hybridized 2.6 times on the average to get acceptable results. The average and standard deviation of several (>3) profiles of each chromosome were calculated, and more profiles were added until the averaged profile and standard deviation did not change after the addition of a new one. When using these criteria, ratios less than 0.85 and larger than 1.15 were never observed in normal versus normal hybridizations. Gains and losses were therefore scored if the ratio was above 1.15 and below 0.85, respectively (DeAngelis et al, 1999).

### DNA flow cytometry

The flow cytometry procedure for determination of tumor-specific S-phase fraction and apoptosis has been described (Stokke et al, 1998a). Briefly, thawed cells were labelled with

© 2001 Cancer Research Campaign phycoerythrin-labelled light chain antibodies for the tumour-characteristic light chain and washed. The cells were thereafter fixed with 1% paraformaldehyde followed by 100% methanol and labelled with FITC-dUTP after incorporation by terminal deoxynucleotidyl transferase for the detection of apoptotic cells, and Hoechst 33258 for assessment of DNA content. After gating away aggregates of cells and apoptotic cells, DNA histograms of the tumour cells and normal cells were obtained by gating on the light chain positive and negative cells, respectively. The fraction of tumour cells was assumed to be equal to the fraction of cells expressing the tumour-characteristic light chain. Ploidy, i.e. DNA index (DI), was calculated as the ratio between the $G_1$ peak positions of the tumour cells and the normal cells.

### Statistical analysis

Relevant statistical tests were performed using the 'SigmaStat' (Jandel Scientific, Erkrath, Germany) and 'SPSS' (SPSS, Chicago, IL) software packages. Relative risks were calculated using Cox uni-and multi-variate regression analysis. Relative risk (RR) is the time-averaged ratio between the fractional survivals in the two groups defined by the assessed variable. *P*-values were calculated using Cox regression analysis and the log-rank test. *P*-values below 0.05 were considered to reflect statistical significance. Cancer-specific survival was determined from the time of the relevant biopsy. Death from any cancer or treatment complications defined an event. Other deaths were censored. The Kaplan-Meier method was used to generate survival curves.

### RESULTS

### CGH aberrations in NHL

Ninety-four cases of B-cell NHL were analysed for chromosomal aberrations by CGH. Table 1 and Figure 1 contain the complete results obtained by CGH, as well as the DNA index for all 94 lymphomas. Regions with a ratio above 1.5 are written in bold print in Table 1 and displayed in bold print in Figure 1. As an example, Figure 2 shows the results obtained for seven of the tumours with respect to chromosomes 1, 3, 9, and 18. Large-scale aberrations, i.e. aberrations detectable by CGH, were found in 80 of 94 tumours (85%), including a majority of the tumours that were found to be DNA diploid by flow cytometry (Table 1). The mean fraction of tumour cells was not different in the samples with and without CGH aberrations (75% for both groups; $P = 0.88$), indicating that the lack of observed aberrations cannot be attributed to a low fraction of tumour cells. The CGH aberrations were non-random, and some of them were local. For classification of an aberration as local, i.e. not encompassing the whole chromosome, we required that at least three tumours defined any border around the 'consensus region'. The two arms of a chromosome were not assumed to be separate entities, and aberrations outside the consensus region(s) on a given

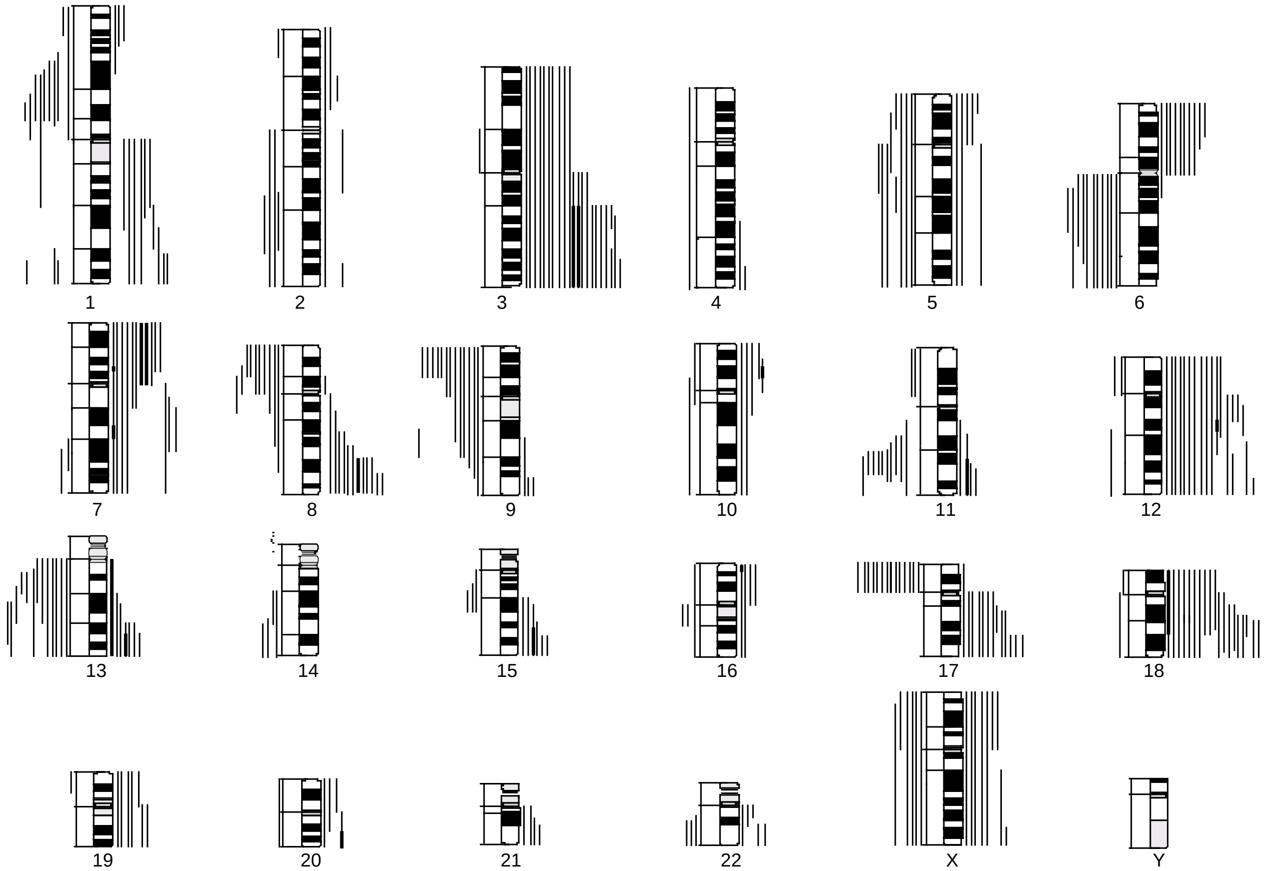

**Figure 1** Chromosome ideogram with losses (left) and gains (right) in 94 cases of NHL. Bold lines represent regions where the green/red fluorescence ratio exceeded 1.5 (`high-level' amplifications).

 © 2001 Cancer Research Campaign

**Table 1** Chromosomal aberrations in 94 cases of B-cell NHL

| Case | Lymphoma subtype (REAL)[a] | Sex | CGH losses | CGH gains[b] | DNA index[c] | No. c-MYC alleles[d] | No. RB1 alleles[d] | No. TP53 alleles[d] |
|---|---|---|---|---|---|---|---|---|
| Small lymphocytic lymphoma | | | | | | | | |
| 140/83 | SLL | M | None | None | 1.00 | 2 | 1 | 2 |
| 086/85 | SLL | M | 11q14–23.1 | None | 1.00 | | 2 | 2 |
| 320/88 | SLL | F | X | None | 1.00 | 2 | 2 | 2 |
| 325/88 | SLL* | M | None | None | 1.00 | | | |
| 416/88 | SLL | M | 17p | 12 | 1.03 | 2 | 2 | 1 |
| 470/88 | SLL | M | None | 10cen-q21.1, 12qcen-13, 12q22-ter | 1.02 | | | |
| 191/89 | SLL | M | None | 12 | 1.02 | 2 | 2 | 2 |
| 339/89 | SLL* | F | 8p21-ter, 11q14–23.1, 13q12.3-ter | 3q, **3q21-ter**, 5p, 7p13-ter, 8q22-ter, 17q21.3-ter | 1.00 | 3 | 1 | 2 |
| 445/89 | SLL | M | None | 2p14–16, 3 | 1.02 | | 2 | |
| 452/89 | SLL | M | 14q24-ter | None | 1.00 | | 2 | |
| 051/90 | SLL | M | 1q41-ter, 17p | 1cen-q31, 3q, 18q12.3-ter, 22q13-ter | 1.05 | 2 | 2 | 1 |
| 159/90 | SLL | F | None | 12 | 1.04 | | | |
| 571/90 | SLL | M | 11q22–23.1 | None | 1.00 | | | |
| 156/91 | SLL* | F | 11q21–23, 15q14–21 | None | 1.00 | 2 | 2 | 2 |
| 244/91 | SLL | M | None | None | 1.00 | 2 | 2 | 2 |
| 462/91 | SLL* | F | 17p | 11q23-ter, 12pter-q15, 13q31-ter, 20p | 1.00 | 2 | 2 | 1 |
| 225/92 | SLL | M | None | 12 | 1.03 | | | |
| 315/92 | SLL | M | None | 12, 15q25-ter | 1.00 | 2 | 1 | 2 |
| 538/92 | SLL | M | 8cen-p21 | 3q21-ter, 8q23-ter | 1.00 | | | |
| Marginal zone lymphoma | | | | | | | | |
| 315/88 | MZL | M | None | 12 | 1.00 | 2 | 1 | 2 |
| 237/91 | MZL* | M | 11q14.3–23 | None | 1.00 | | | |
| 448/91 | MZL | M | None | 3, 18, 19 | 1.05 | | 2 | 2 |
| Follicle centre lymphoma | | | | | | | | |
| 377/83 | FCL I/II | M | None | 7pter-q12 | 1.00 | 2 | 2 | 2 |
| 364/86 | FCL I/II + SLL | F | 19p13.2-ter | 18pter-q21 | 1.02 | | | |
| 369/86 | FCL I* | F | None | None | 1.02 | | | |
| 021/87 | FCL I/II | M | None | 18pter-q21.1 | 1.00 | | | |
| 241/87 | FCL I/II | F | None | None | 1.02 | | 2 | 2 |
| 345/87 | FCL II | F | None | 18q | 1.00 | | | |
| 018/88 | FCL II | F | None | 1p21–22, 13q21-ter | 1.00 | 2 | 2 | 2 |
| 041/88 | FCL II | F | None | 18q12.3-ter | 1.03 | | | |
| 046/88 | FCL II* | F | None | None | 0.97 | 2 | 2 | 2 |
| 047/88 | FCL I/II* | M | 6cen-q22, 13q13–21.1 | None | 1.00 | 2 | 1 | 2 |
| 176/88 | FCL II | F | None | 7, 8q21.3-ter, 18q12–22 | 1.04 | | | |
| 233/88 | FCL II | F | 6q14-ter, 13cen-q22 | None | 1.00 | 2 | 1 | 2 |
| 275/88 | FCL I/II | M | None | None | 1.00 | 2 | 2 | 2 |
| 284/88 | FCL II | F | None | 20pter-q12, 22cen-q12 | 1.02 | 2 | 2 | 2 |
| 381/88 | FCL II | M | None | 18, X | 1.03 | | | |
| 399/88 | FCL III | M | 6cen-q24, 17p | 1p34.3-ter, 8q24-ter, 17q, 22q | 1.00 | 2 | 2 | 1 |
| 468/88 | FCL I | M | None | 15q25-ter | 1.00 | | | |
| 064/89 | FCL II* | F | 1cen-p22.2 | 1q32-ter, 6pter-q15, 12pter-q21, **12q14**, 13q31-ter, **13q32-ter**, 16, 20q, **20q13**, 21q22 | 1.26 | 2 | 2 | 3 |
| 287/89 | FCL I | M | 6q14–22.1 | 2 | 1.03 | | | |

© 2001 Cancer Research Campaign

British Journal of Cancer (2001) **85**(12), 1900–1913

(Table 1 Continued)

| | | | | | | | | |
|---|---|---|---|---|---|---|---|---|
| 311/89 | FCL II | F | None | 7q21–31, 13q32-ter, X | 1.05 | 2 | 2 | 2 |
| 521/89 | FCL II | F | None | 17q24-ter, 20, 21q | 1.00 | | | |
| 140/90 | FCL II* | M | None | 1q41-ter, 17, 18 | 1.03 | | | |
| 372/90 | FCL II* | F | 17p | 1q41-ter, 7p13-ter | 1.07 | | | |
| 377/90 | FCL II | F | None | 2cen-q22, 2q36-ter | 1.06 | | 2 | 2 |
| 416/90 | FCL II | M | None | None | 1.00 | | 2 | 2 |
| 577/90 | FCL II | M | 5, 9p, 17p | 3, 12, X | 1.97 | | | |
| 581/90 | FCL II* | F | 17p | 1q,3,5,7,10,12q,17q,18q12.3–21,X | 1.22 | | | |
| 635/90 | FCL I/II* | M | None | None | 1.00 | | | |
| 382/91 | FCL II* | M | 16cen-q13 | 17q | 1.02 | 2 | 2 | 2 |
| 436/91 | FCL II | M | None | 18, **18pter-q21** | 1.04 | | | |
| 489/91 | FCL I/II* | M | None | 12q13–15,17q21–22 | 1.03 | | | |
| 533/91 | FCL II | F | None | None | 1.00 | | | |
| 021/92 | FCL II | F | 16cen-q13,X | 12q24.2-ter,17q | 1.02 | | | |
| 103/92 | FCL II* | M | 1p35-ter | 1q | 1.00 | | 2 | 2 |
| 130/92 | FCL I/II* | M | None | 12cen-q13,16p,18,19 | 1.03 | | | |
| 300/92 | FCL II | M | 17p | 3q24-ter | 1.00 | | 2 | 2 |
| Mantle cell lymphoma | | | | | | | | |
| 123/84 | MCL* | M | 9p21-ter,11q23-ter,13q21.3-ter, 22q12.2-ter | 3q22-ter,10cen-p13, **10p12**,Xq27-ter | 1.04 | | 2 | |
| 358/87 | MCL* | F | 2q,6q,8p21-ter,9pter-q33, 11p12-ter, 13q,14q21-ter | 3,6p,7,8q21.2-ter,15q21-ter | 1.94 | | 3 | |
| 154/88 | MCL | M | 1p21–31,5p14-q13.1,8p,9,12q 15-ter,13q | 1p34-ter,16,**16p13.3**,19,22cen-12.1 | 0.95 | 2 | 1 | 2 |
| 265/88 | MCL* | F | 1p,9p21-ter,11p12-ter, 18p | 11q13-ter,18q12-ter | 1.00 | 2 | 2 | 2 |
| 010/89 | MCL* | M | 1p21–31.1,9p21-ter,11q21–23.1, 13cen-21.1,18p | 3q21-ter | 1.00 | | 1 | 2 |
| 129/89 | MCL* | F | 3cen-p14,8p,11q14–23 | 3q21-ter,4q33-ter,5p,8q22-ter | 1.02 | 3 | 2 | 2 |
| 309/89 | MCL* | M | 1p13.3–31,6q,9p21-ter,13q, 14q21–24.1,22q12.2-ter | 6p | 1.83 | 3+4 | 2+3 | 3 |
| 472/90 | MCL | F | None | 2p13-ter,5,6p22-ter,7p,**7p**,8q23-ter,**8q23-ter**,10p12-ter, 12,17q24-ter,18pter-q21 | 1.16 | | 2 | 2 |
| 383/91 | MCL | M | 9pter-q22,13q | 9q34-ter | 0.97 | | 1 | |
| 428/91 | MCL | M | 1p31.1-q25 | None | 2.00 | 4 | 4 | 4 |
| 037/92 | MCL* | F | 7q31–32,9p,17p | 17q24-ter,21q21-ter | 1.00 | 2 | 2 | 1 |
| 067/92 | MCL* | M | 1p21,8p,9pter-q31,11q22–23.1, 13q,22q12-ter | 3q21–23,3q26-ter,8q,15q21.2-ter, **15q24-ter** | 0.98 | 3 | 1 | 2 |
| 245/92 | MCL* | M | None | 3q, **3q21-ter** | 1.03 | | 2 | 2 |
| Diffuse large B-cell lymphoma | | | | | | | | |
| 122/84 | DLBCL | F | 1p21–31.1,2p23-ter,2q23-ter, 2q23–33,5q,6q,8pter-q21,15cen-q21,17p | 7,**7p13,7q22**,11q23.3-ter,16p | 1.23 | 2 | 2 | 1 |
| 255/85 | DLBCL | F | 6q,10pter-q11,12p | 3,6p,7p,**7p**,18q21-ter | 1.04 | | 2 | 2 |
| 377/87 | DLBCL* | M | 13q13–14 | None | 1.03 | | 1 | 2 |
| 390/87 | DLBCL | M | 18q | 1q,11q14-ter,**11q22.2-ter** | 1.00 | 2 | 2 | 2 |
| 399/87 | DLBCL | F | X | 1p31.2-ter,5q,12, 15q23-ter,17q21.3-ter,19q | 1.06 | 2 | 2 | 2 |
| 258/88 | DLBCL* | M | 5,17p | 6p,8q23-ter,12q21.3-ter,13q31-ter,21,Xp | 1.07 | 3+4 | 2+4 | 1+2 |
| 287/88 | DLBCL* | F | 4,17p,18p | 8cen-p12,12,15q21-ter,18cen-q22,Xp | 1.00 | | | |
| 454/88 | DLBCL | F | 6q,11q13-ter,17p | 10,17q,22q | 1.02 | | 2 | 1 |
| 131/89 | DLBCL | M | 1p21–32,8p12-q12 | 3q,7pter-q11,8q23-ter,9q22-ter,16p,18 | 2.23 | 8 | 4 | 4 |
| 438/89 | DLBCL | F | 2q24–34,14q24.3-ter | None | 1.00 | 2 | 2 | 2 |

*© 2001 Cancer Research Campaign*

(Table 1 Continued)

| | | | | | | | | |
|---|---|---|---|---|---|---|---|---|
| 525/89 | DLBCL | F | 1q42-ter,5cen-q14,9q21.3–22,Xp | 1cen-q31,3,6p,18q21-ter,Xq21-ter | 1.07 | 2 | 2 | 2 |
| 034/90 | DLBCL + SLL | M | 1q42-ter,15q15–21 | 1cen-q25,3,7p,9q33-ter,22q13-ter | 1.16 | | 3 | |
| 612/90 | DLBCL | F | 9pter-q22 | 3q26.3-ter, 8q24-ter,19p | 1.04 | 3 | 2 | 2 |
| 340/91 | DLBCL | M | None | 7q | 1.03 | | | |
| 070/92 | DLBCL | F | None | None | 1.01 | 2 | 2 | 2 |
| 117/92 | DLBCL* | F | 5 | 1q31–33,3, 6p21.3-ter,13q21.3-ter, X | 1.00 | 2 | 2 | 2 |
| 050/93 | DLBCL | M | 5q,8pter-q23,9pter-q21,13q21.3–32, 17p,20 | 3,17q,19 | 1.00 | 2 | 2 | 1 |
| Burkitt's and Burkitt-like lymphoma | | | | | | | | |
| 458/88 | BL-like | F | 9p21-ter,10p11-qter,16,X | 5p15,8q13-ter,13q,**13q**,19q | 1.03 | 3 | 0 | 2 |
| 277/89 | BL | F | None | None | 1.04 | 2 | 2 | 2 |
| 042/92 | BL-like | M | None | None | 1.03 | 2 | 2 | 2 |
| Unclassified B-cell lymphoma | | | | | | | | |
| 214/88 | | M | 6q | 6q | 1.00 | 2 | 2 | 2 |
| 537/91 | | F | None | None | 1.00 | | | |
| 008/92 | | F | 2q,5p14-ter,5q14–21,6q,7q3 1.3-ter,8pter-q12,12p,13q 14.3–22,Xp22.1-qter | 4q28-ter,6p,7q11.23–31.1,18 | 1.00 | | 2 | 1 |

[a]Rebiopsy cases are labelled with a '*'. [b]Regions where the green/red ratio exceeded 1.5 ('high-level' amplifications) are repeated in bold. [c]The DNA index was measured by flow cytometry as described in 'Materials and methods'. Cases with a DNA index between 0.93 and 1.07 were considered DNA diploid, since only one $G_1$ peak could be identified in the ungated DNA histograms. DNA tetraploid cases have a DNA index in the region 1.9–2.1. [d]Data were taken from Galteland et al (1999) or assessed as described therein.

chromosome were assumed to have arisen as a consequence of the gain or loss of important gene(s) within the consensus region. The most frequent (≥ 10%) losses were found at 1p21–31.1 (9 cases, 10%), 6cen-q24 (11 cases, 12%), 8p (10 cases, 11%), 9p21-ter (13 cases, 14%), 11q21–23.1 (10 cases, 11%), 13q13–21.1 (11 cases, 12%), and 17p (14 cases, 15%). The gains were clustered at 3q21-ter (21 cases, 23%), 6p (10 cases, 11%), 7p (11 cases, 12%), 8q23-ter (12 cases, 13%), 12cen-q15 (16 cases, 17%), 17q24-ter (12 cases, 13%), and 18q13.3–21 (19 cases, 20%).

### Verification of CGH results by direct measurements of specific gene copy numbers

The copy numbers of the c-MYC, RB1, and TP53 genes were determined by interphase fluorescent in situ hybridization (FISH, Galteland et al, 1999; data for 44 cases were taken from the reference). These copy numbers are given in Table 1 and show that CGH detected gains at 8q24 in all cases where the FISH-detected c-MYC copy number was increased relative to the DNA index. Three plus 4 and 4 copies of c-MYC were found in cases 309/89 and 428/91, respectively, but no 8q gain was detected by CGH, which is in agreement with the high DNA index of these tumours. The gain of 8q24-ter by CGH observed in cases 399/88, with only 2 copies of the c-MYC gene, could be due to amplification of the telomeric region distal of c-MYC. There was also good agreement between the CGH results on 17p and the number of TP53 copies (Table 1; see also Stokke et al, 2000). The exceptions were case 300/92 with loss of 17p by CGH but 2 TP53 copies, and case 8/92 with normal 17p by CGH and only 1 TP53 copy. Four of 12 cases with loss of RB1 did not show any loss at 13q by CGH. Equally interesting, case 458/88 showed high-level gain (i.e. ratio above 1.5) of chromosome 13 by CGH, while no RB1 copies could be detected by FISH. Case 458/88 was the only one in this panel of tumours which did not express pRB by immunoblotting and immunohistochemistry (unpublished results). The three other cases with 1 RB1 copy, but no losses on 13q by CGH, were small lymphocytic lymphomas. Cases 123/84, 8/92, and 50/93 had 2 RB1 copies, in agreement with the CGH results that showed a deletion telomeric of RB1.

### Frequency distribution of CGH aberrations

The frequency distribution of the number of aberrations was bimodal, with a natural cut-off between the groups at approximately four aberrations (Figure 3). Attempts to fit this distribution to a single Poisson distribution failed ($P < 0.001$). A linear combination of two Poisson distributions with average values of 1.5 and 7.5, respectively, gave significant fit ($R^2 = 0.94$, $P = 0.75$). This shows that two groups of lymphomas can be defined, one with a low number of aberrations (0–3), and one with a high number of aberrations (≥ 4).

### Associations between CGH aberrations

Table 2 shows that several of the aberrations tended to occur together. Loss of 8p was correlated with seven other aberrations; partially reflecting that 9 of 10 tumours with 8p loss had a high number of aberrations. The deletions on 1p21–31.1, 8p, 9p21-ter, and 13q13–21.1, frequently found in mantle cell lymphomas (see below), showed pairwise associations. The loss of 8p, and the gains of 3q21-ter, 7p, and 8q23-ter were also pairwise correlated. Three chromosomes (6, 8, and 17) had frequent loss of one arm and gain of

© 2001 Cancer Research Campaign

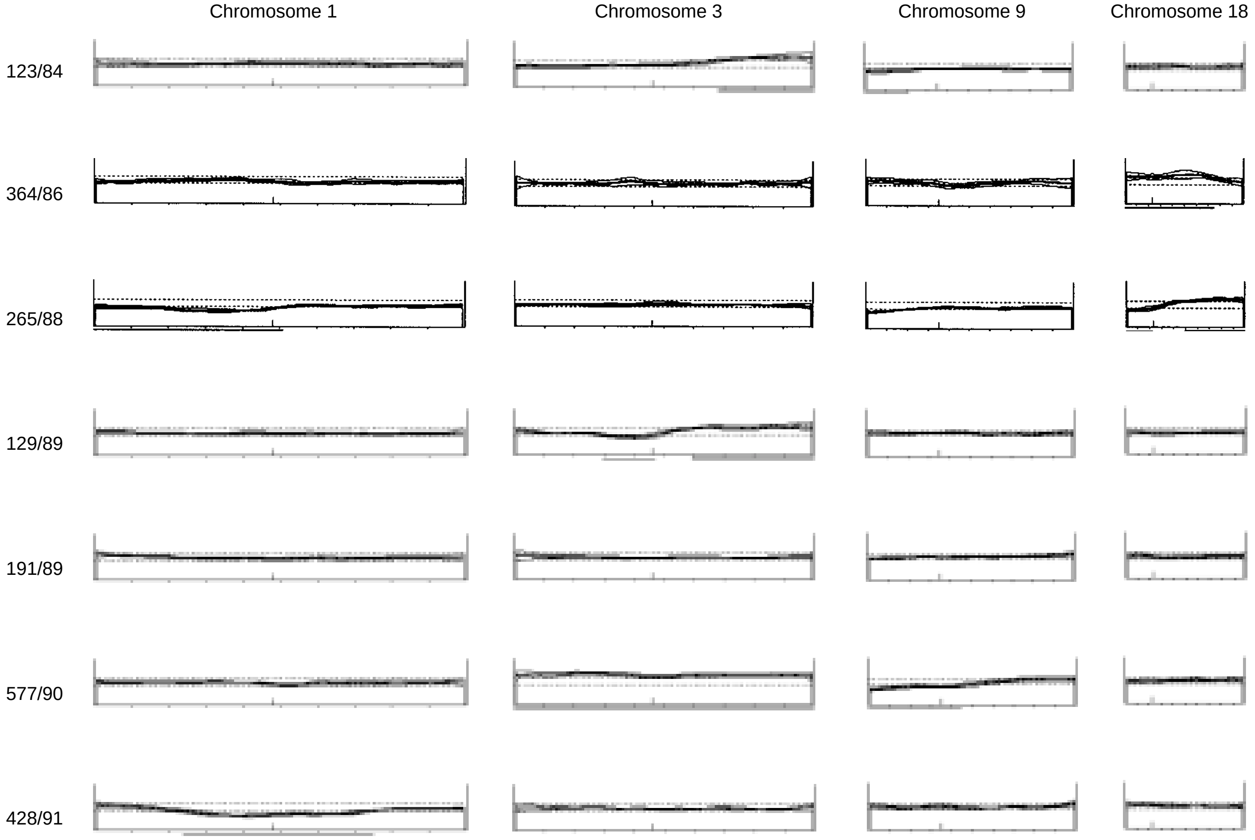

**Figure 2** Averaged ratios and standard deviations for chromosomes 1, 3, 9, and 18 for 7 NHL cases. Case number is indicated to the left. The horizontal dotted lines at 0.85 and 1.15 represent the cut-offs established from normal hybridizations for definition of losses (indicated by thin lines below the chromosome fractional length axis) and gains (indicated by bold lines), respectively.

the other (Figure 1). For these chromosomes, the loss of one arm was associated with gain of the other (Table 2). Gain of 12cen-q15 was negatively correlated with the majority of the other aberrations, but none of these negative associations were statistically significant.

Gain of 12cen-q15 and 18q13.3–21 were the only aberrations which were found in more than 10% of the cases with a low number (0–3) of aberrations. The frequencies of these aberrations were less than two-fold higher among the cases with a high number of aberrations ($P$ = 0.25 and 0.11, respectively). The 11q21–23.1 deletion was found 2.7 times more frequently in the lymphomas with many aberrations compared to the ones with few, but the difference was not significant ($P$ = 0.16). However, while the loss of 11q21–23.1 occurred together with several other aberrations in 4 MCL cases, three of the 4 losses at 11q21–23.1 in SLL were found in tumours with a low number of aberrations. For the remaining CGH aberrations, the frequency in the tumours with many aberrations was >3.2 times higher than in the cases with few aberrations ($P$ < 0.05 in all cases).

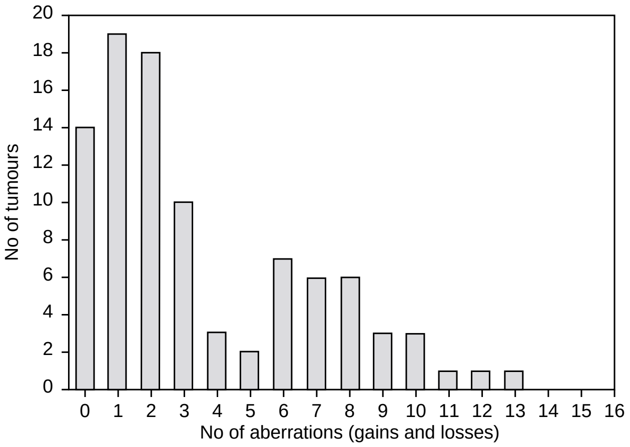

**Figure 3** Histogram of the total number of CGH aberrations in each tumour. The number of tumours was plotted as a function of the number of aberrations, i.e. the sum of the number of losses and gains. The distribution could be fitted to a sum of two Poisson-distributions with average values of 1.5 and 7.5 ($R^2$ = 0.94, $P$ = 0.75), but no significant fit was obtained with a single Poisson-distribution ($P$ < 0.001; $\chi^2$ test).

### Lymphoma subtype and CGH aberrations

A high number of CGH aberrations was associated with MCL and DLBCL ($P$ < 0.001). Figure 4 shows the distribution of the aberrations according to lymphoma sub-types. Loss of 1p21–31.1 ($P$ < 0.001), 8p ($P$ = 0.02), 9p21-ter ($P$ < 0.001), 11q21–23.1 ($P$ = 0.03), and 13q13–21.1 ($P$ < 0.001) occurred more frequently in MCL than in the other subtypes. Gain of 6p ($P$ = 0.04) was associated with DLBCL, and gain of 12cen-q15 was found more frequently in SLL than in the other lymphoma groups ($P$ = 0.04).

### The impact of CGH aberrations on cellular phenotypes

The tumour-specific S phase and apoptotic fractions of the tumours studied here have been published earlier (Stokke et al, 1998a). A high number of CGH aberrations ($P$ < 0.001; Mann-Whitney), as well as loss of 8p ($P$ = 0.03) and 17p ($P$ = 0.005), and

 © 2001 Cancer Research Campaign

**Table 2** Matrix of correlation coefficients for CGH aberrations[a]

| | −1p21-31.1 | −6cen-q24 | −8p | −9p21-ter | −11q21-23.1 | −13q13-21.1 | −17p | +3q21-ter | +6p | +7p | +8q23-ter | +12cen-q15 | +17q24-ter |
|---|---|---|---|---|---|---|---|---|---|---|---|---|---|
| −1p21-31.1 | 1.0 | | | | | | | | | | | | |
| −6cen-q24 | 0.11 (.30) | 1.0 | | | | | | | | | | | |
| −8p | **0.36**(.001) | 0.20 (.06) | 1.0 | | | | | | | | | | |
| −9p21-ter | **0.39** (.000) | 0.05 (.66) | **0.26**(.01) | 1.0 | | | | | | | | | |
| −11q21-23.1 | 0.12 (.24) | −0.02 (.86) | **0.22** (.04) | 0.16 (.12) | 1.0 | | | | | | | | |
| −13q13-21.1 | **0.33** (.001) | **0.38** (.000) | **0.41** (.000) | **0.43** (.000) | 0.20 (.06) | 1.0 | | | | | | | |
| −17p | −0.04 (.74) | 0.13 (.22) | 0.05 (.63) | 0.09 (.38) | −0.05 (.65) | −0.15 (.14) | 1.0 | | | | | | |
| +3q21-ter | 0.09 (.41) | −0.04 (.73) | **0.40** (.000) | **0.30** (.003) | **0.23** (.03) | 0.12 (.24) | 0.13 (.20) | 1.0 | | | | | |
| +6p | 0.12 (.24) | **0.41** (.000) | 0.11 (.31) | 0.06 (.55) | −0.12 (.25) | 0.20 (.06) | −0.05 (.65) | 0.15 (.16) | 1.0 | | | | |
| +7p | 0.11 (.30) | 0.18 (.09) | **0.30** (.003) | −0.05 (.63) | −0.02 (.86) | 0.07 (.48) | 0.13 (.22) | **0.28** (.007) | 0.20 (.06) | 1.0 | | | |
| +8q23-ter | 0.09 (.37) | 0.06 (.57) | **0.49** (.000) | **0.22** (.04) | 0.18 (.09) | 0.16 (.13) | 0.02 (.85) | **0.33** (.001) | 0.18 (.09) | **0.36** (.001) | 1.0 | | |
| +12cen-q15 | −0.05 (.62) | −0.17 (.11) | −0.16 (.13) | −0.10 (.33) | −0.16 (.13) | −0.17 (.11) | **0.21** (.05) | −0.11 (.30) | 0.03 (.79) | 0.01 (.91) | −0.09 (.39) | 1.0 | |
| +17q24-ter | −0.12 (.23) | 0.06 (.57) | 0.08 (.47) | 0.03 (.76) | 0.08 (.47) | −0.04 (.70) | **0.29** (.006) | 0.02 (.81) | −0.03 (.78) | 0.16 (.13) | 0.14 (.18) | 0.08 (.44) | 1.0 |
| +18q13.3-21 | 0.02 (.88) | −0.02 (.86) | −0.02 (.99) | −0.13 (.23) | −0.17 (.09) | −0.10 (.33) | 0.01 (.92) | 0.11 (.28) | 0.17 (.10) | **0.23** (.03) | 0.05 (.66) | 0.05 (.60) | 0.05 (.66) |

[a]The correlation coefficients were calculated using Kendall's tau-b test. *P* values are given in parentheses. Significant results are in bold print.

gain of 3q21-ter ($P < 0.001$), 6p ($P = 0.02$), 7p ($P = 0.007$), and 8q23-ter (0.01) were associated with a high S phase fraction. None of the CGH aberrations were associated with apoptotic fraction ($P \geq 0.13$).

### CGH aberrations and patient survival

The prognostic values of the different gains and losses are given in Table 3. The relative risks associated with a high number of CGH aberrations, loss of 6cen-q24, 8p, 9p21-ter, 11q21–23.1, 13q13–21.1 and 17p, and gain of 3q21-ter and 6p, were significantly higher than one. Multivariate survival analysis revealed that the only independent prognostic factors were loss of 11q21–23.1 and 17p, and gain of 6p (Table 3).

The CGH parameters with independent prognostic value were analysed by Cox multivariate regression analysis together with lymphoma sub-type, as well as clinical and other biological parameters (Table 4). All parameters except for treatment had significant prognostic value in univariate analysis (Table 4), but only –11q21–23.1, –17p, +6p, and IPI had independent prognostic value. S phase fraction had marginal significance in multivariate analysis; further investigations have shown that the patients with the CGH aberrations with independent prognostic value survive a shorter time if the tumour S phase fraction is higher than 3% (data not shown). The survival was similar for the patients receiving different treatments (chemotherapy without doxorubicin, chemotherapy with doxorubicin, 'other' treatments; $P > 0.75$ for pairwise comparisons), and it will be demonstrated later (Figure 5) that the prognostic value of the CGH parameters and IPI is high in all these three treatment groups.

In an attempt to construct a combined parameter, we noticed that the –11q21–23.1, –17p, and +6p CGH parameters were negatively correlated (Table 2), which was not unexpected in view of their independent prognostic value (Tables 3, 4). The most logical way of combining these parameters would therefore be to include cases in the 'poor survival' group if they had any of these aberrations (and/or). This CGH combination parameter (Figure 5A) was substituted in an analysis similar to the one shown in Table 4 instead of the 3 individual CGH aberrations, and was the only parameter together with IPI which came out with significant prognostic value (data not shown). In a similar fashion as above, a single binary combined 'CGH/IPI' parameter was constructed such that cases were allocated to the 'poor survival' group if they had any of the three prognostic CGH aberrations and/or a high IPI score (i.e. IPI > 2). The prognostic value of the combined CGH/IPI parameter is shown in Figure 5C. When this parameter was entered into the multivariate analysis as shown in Table 4 instead of the CGH aberrations and IPI, it emerged as the only significant parameter, while S phase again had marginal significance (data not shown). This 'backtesting' of the combined parameter in multivariate analysis strongly suggests that we have chosen the best way (with this simple approach) of combining the individual parameters with independent prognostic value.

The survival according to the combined CGH/IPI parameter is shown for patients receiving different treatments (Figures 5D, E, F), with different lymphoma subtypes (Figures 6A, B, C, D), for the patients assessed at the first presentation (Figure 6E), as well as for the patients with relapsed NHL (Figure 6F). CGH/IPI had high prognostic value in all these sub-groups ($P \leq 0.006$).

*© 2001 Cancer Research Campaign*

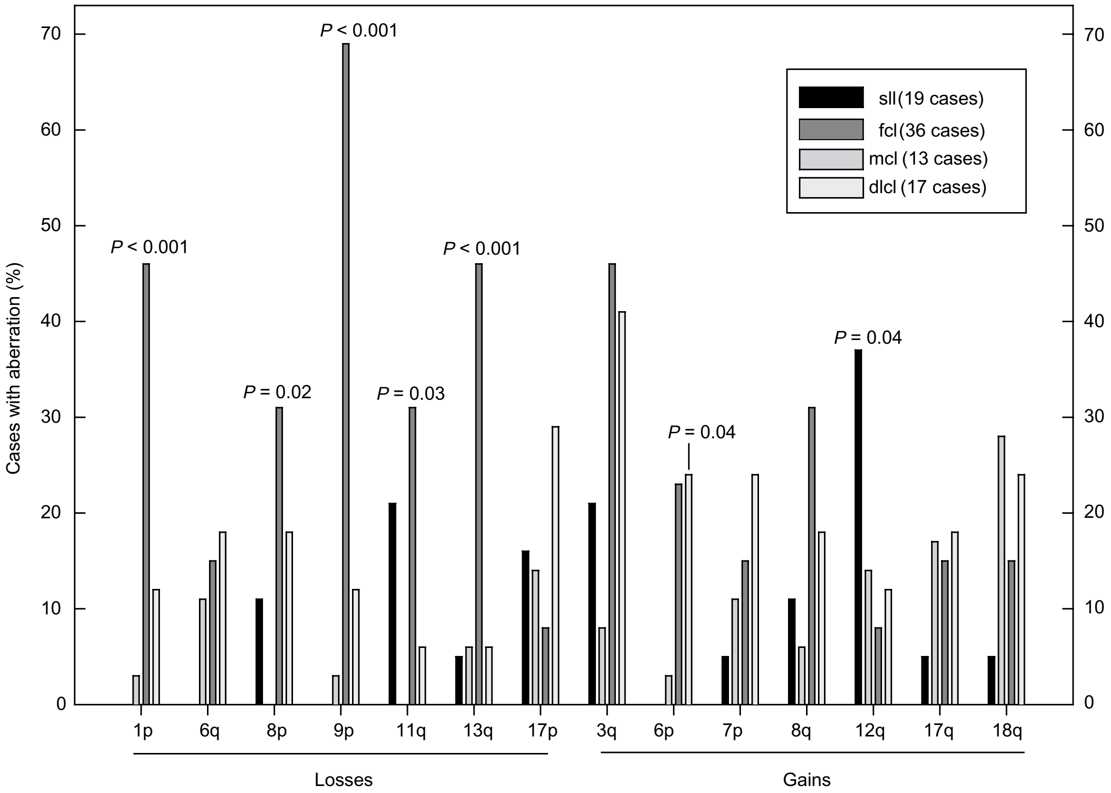

**Figure 4** Distribution of the most frequent CGH aberrations according to lymphoma subtype. The figure shows the frequency of the aberrations, which were found in nine NHL cases or more (≥10%), within each lymphoma sub-type.

**Table 3** Prognostic values of CGH aberrations in B-cell NHL[a]

| Aberration (No. of cases with aberration) | Univariate analysis: Relative risk[b] | Univariate analysis: 95% Confidence interval | Univariate analysis: *P*-value[c] | Multivariate analysis[d]: Relative risk[b] | Multivariate analysis[d]: 95% Confidence interval | Multivariate analysis[d]: *P*-value |
|---|---|---|---|---|---|---|
| –1p21–31.1 (9) | 1.7 | 0.78–3.9 | 0.18 | | | 0.80 |
| –6cen-q24 (11) | **2.1** | 1.1–4.2 | 0.04 | | | 0.64 |
| –8p (10) | **3.3** | 1.6–6.8 | 0.002 | | | 0.27 |
| –9p21-ter (13) | **2.1** | 1.1–4.0 | 0.02 | | | 0.67 |
| –11q21–23.1 (10) | **3.8** | 1.9–7.8 | < 0.001 | **6.8** | 3.1–15 | < 0.001 |
| –13q13-21.1 (11) | **2.3** | 1.2–4.5 | 0.01 | | | 0.57 |
| –17p (14) | **4.4** | 2.2–8.6 | < 0.001 | **7.2** | 3.5–15 | < 0.001 |
| +3q21-ter (21) | **1.9** | 1.0–3.5 | 0.04 | | | 0.52 |
| +6p (10) | **4.2** | 2.0–8.8 | < 0.001 | **8.8** | 3.9–20 | < 0.001 |
| +7p (11) | 1.3 | 0.60–3.0 | 0.47 | | | |
| +8q23-ter (12) | 1.9 | 0.95–3.8 | 0.07 | | | 0.92 |
| +12cen–q15 (15) | 0.99 | 0.42–2.3 | 0.98 | | | |
| +17q24-ter (12) | 1.3 | 0.58–2.9 | 0.54 | | | |
| +18q13.3–21 (19) | 1.0 | 0.54–2.0 | 0.94 | | | |
| #CGH aberrations > 3 (33) | **3.7** | 2.1–6.6 | < 0.001 | | | 0.38 |

[a]Survival data were available for 92 patients. [b]Relative to the cases without the respective aberration. Cox regression analysis was employed. Significant values are in bold print. [c]From Cox regression analysis. The log-rank test gave p-values with less than 15% deviation from the values in the table. [d]Aberrations with *P*<0.20 in univariate analysis were included in Cox forward and backward multivariate regression analysis (identical results).

### DISCUSSION

Chromosomal aberrations have high prognostic value and are used for treatment stratification in acute lymphoblastic leukemia (Pui and Evans, 1998). Intensive combination chemotherapy can cure almost half of the patients with aggressive NHL (e.g., Fisher et al, 1993). It is therefore important to establish reliable prognostic factors for treatment stratification of NHL patients with large differences with respect to survival. Traditionally, histology and stage have been used as the criteria for selection of NHL patients for different types of therapy. Clinical parameters (IPI, The International Non-Hodgkin's Lymphoma Prognostic Factors Project, 1993), and tumour cell proliferation (Stokke et al, 1998b and references therein) are known prognostic factors in B-cell NHL, which, however, have not yet been much used for treatment stratification. In this retrospective study, we have identified three chromosomal aberrations with high and independent prognostic value in B-cell NHL. Loss of 11q21–23.1 and 17p, and gain of 6p

 *© 2001 Cancer Research Campaign*

**Table 4** Prognostic values of CGH aberrations, biological and clinical parameters in B-cell NHL[a]

| Aberration (No. of cases with aberration or phenotype) | Univariate analysis | | | Multivariate analysis[d] | | |
|---|---|---|---|---|---|---|
| | Relative risk[b] | 95% Confidence interval | *P*-value[c] | Relative risk[b] | 95% Confidence interval | *P*-value |
| –11q21–23.1 (10) | **3.8** | 1.9–7.8 | <0.001 | **10** | 4.3–23 | <0.001 |
| –17p (14) | **4.4** | 2.2–8.6 | <0.001 | **5.0** | 2.4–11 | <0.001 |
| +6p (10) | **4.2** | 2.0–8.8 | <0.001 | **6.7** | 2.9–16 | <0.001 |
| S phase fraction > 3% (30) | **3.1** | 1.8–5.6 | <0.001 | | | 0.05 |
| IPI > 2 (25) | **3.9** | 2.1–7.1 | <0.001 | **3.7** | 1.8–7.5 | <0.001 |
| Lymphoma subtype FCL (36)[e] | **0.41** | 0.22–0.75 | 0.004 | | | 0.07 |
| Relapse (vs. primary) biopsy (30) | **2.3** | 1.4–4.1 | 0.002 | | | 0.20 |
| cyclin D1 expression (14) | **2.4** | 1.2–4.6 | 0.01 | | | 0.17 |
| t(14;18) (37) | **0.53** | 0.29–0.94 | 0.03 | | | 0.35 |
| B symptoms (22) | **2.7** | 1.5–4.9 | 0.001 | | | 0.19 |
| Treatment[f] | | | >0.75 | | | |

[a]Survival data were available for 92 patients. [b]Relative to the cases without the respective aberration or phenotype. Cox regression analysis was employed. Significant values are in bold print. [c]From Cox regression analysis. The log-rank test gave *P*-values with less than 15% deviation from the values in the table. [d]Aberrations with *P* < 0.20 in univariate analysis were included in Cox forward and backward multivariate regression analysis (identical results). [e]FCL patients (median survival: 83 months) survived longer than the other patients (median survival: 33 months). There were no significant differences between the survival in the other lymphoma subtypes (*P* > 0.12). [f]Patient treatments were essentially chemotherapy with (39 cases), or without (35 cases) doxorubicin, or other types of treatment (18 cases; interferon, radiation). The survival was not different in these 3 groups (*P* > 0.75 for all 3 pairwise comparisons; log-rank test).

were all associated with relative risks larger than 5. The International Prognostic Index was the only other parameter with significant independent prognostic value, and a simple binary combination parameter of the three CGH aberrations and IPI had high prognostic value for groups of patients with various lymphoma sub-types, having received different treatments, and presenting with primary or relapse disease.

In agreement with earlier studies, we found a number of recurrent losses and gains common to several sub-types of B-cell NHL. CGH is a relatively new technique, and the sensitivity and specificity of the method in terms of absolute copy numbers of specific loci have only been documented in a few reports (Kallionemi et al, 1994b; Bentz et al, 1995, 1996; Tirkkonen et al, 1998). For 64 of the cases reported in this study, we have also performed interphase FISH to directly assess the copy number of the c-MYC, RB1, and TP53 gene loci. The results obtained with c-MYC and TP53 document that, at least when employing the criteria described here, CGH is sensitive enough to detect gain and loss of one copy, respectively. However, this sensitivity depends on a predominance of tumor cells in the sample (Kallioniemi et al, 1994a). Four of 12 cases with RB1 deletion by FISH did not show a reduced ratio at 13q14 by CGH. This may imply a poor sensitivity of CGH at this locus, but it is more likely that the deleted regions around RB1 are so small in many cases that CGH is not able to detect them. The specificity of CGH appears to be high, since only one case without FISH aberration was found to have CGH aberration at the corresponding locus.

Twelve of the frequent (>10% of cases) CGH aberrations reported here have been observed in NHL before (Bentz et al, 1995, 1996, 2000; Monni et al, 1996, 1998; Dierlamm et al, 1997; Autio et al, 1998; Beà et al, 1999). Seven of these 12 aberrations, the losses at 6cen-q24, 11q21–23.1, and 13q13–21.1, and the gains at 3q21-ter, 7p, 12cen-q15, and 18q13.3–21, are frequent findings also in classical cytogenetic studies (Heim and Mitelman, 1995). Moreover, banding techniques have revealed frequent rearrangements involving the 5 other regions (1p, 6p, 9p, 8q, and 17p) in B-cell NHL (Heim and Mitelman, 1995). CGH studies in NHL indicate that the rearrangements at the latter five chromosomal regions are not balanced (this work; Bentz et al, 1995; 1996; 2000; Monni et al, 1996, 1998; Dierlamm et al, 1997; Beà et al, 1999). Some of the aberrations were local in the majority of cases where they occurred. Our data defined 1p21–31.1 as a consensus region for the losses at 1 p, in excellent agreement with the results of others (Monni et al, 1996, 1998; Beà et al, 1999; Bentz et al, 2000). Interestingly, an almost identical consensus region for the losses at 1 p was found in breast (Tirkkonen et al, 1998) and colorectal carcinomas (DeAngelis et al, 1999). The losses at 11q were also mostly local, with a consensus region at 11q21–23.1, again in perfect agreement with the minimal common region of deletion determined by others (Monni et al, 1998; Beà et al, 1999; Bentz et al, 2000).

Novel findings of this work include the frequent loss of 8p, and the gain of 17q24-ter. The loss of 8p was associated with the gain of 8q (Table 2). The 17q gains were associated with 17p losses, and may be the result of formation of an isochromosome 17q in some cases.

Suggested candidates for tumour suppressor genes and proto-oncogenes at the chromosomal regions which are frequently lost and gained, respectively, include the ATM gene at 11q23.1 (Bullrich et al, 1999; Schaffner et al, 2000), the TP53 gene at 17p13.1 (Stokke et al, 2000), and the BCL-2 gene at 18q21 (Monni et al, 1997). Other tumour suppressor genes and proto-oncogenes, which are located within the frequently altered regions, include p16INK4a (9p21), RB-1 (13q14), BCL6 (3q27), CCND3 (6p), and c-MYC (8q24). We have investigated the expression of p16, pRB, bc16, cyclin D3, and c-myc by immunoblotting and immunohistochemistry in the tumours presented in this paper (unpublished). Additionally, we have determined the methylation status of the p16INK4a gene (E Hovig and T Stokke, unpublished). We did not find any decreased expression of p16 and pRB in the tumors with 9p21-ter and 13q13–21.1 (RB1) deletions, respectively, compared to the others. Case 458/88 was the only case that did not express pRB; this case had high-level amplification of 13q by CGH, but no RB-1 alleles (Galteland et al, 1999). Also, none of 13 cases with 9p21-ter loss by CGH had detectable methylation of the p16INK4 gene. The cases with gain of 3q21-ter, 6p, and 8q23-ter did not show enhanced expression of bc16, cyclin D3, and c-myc, respectively, compared to the others. Hence, the identities

*© 2001 Cancer Research Campaign*

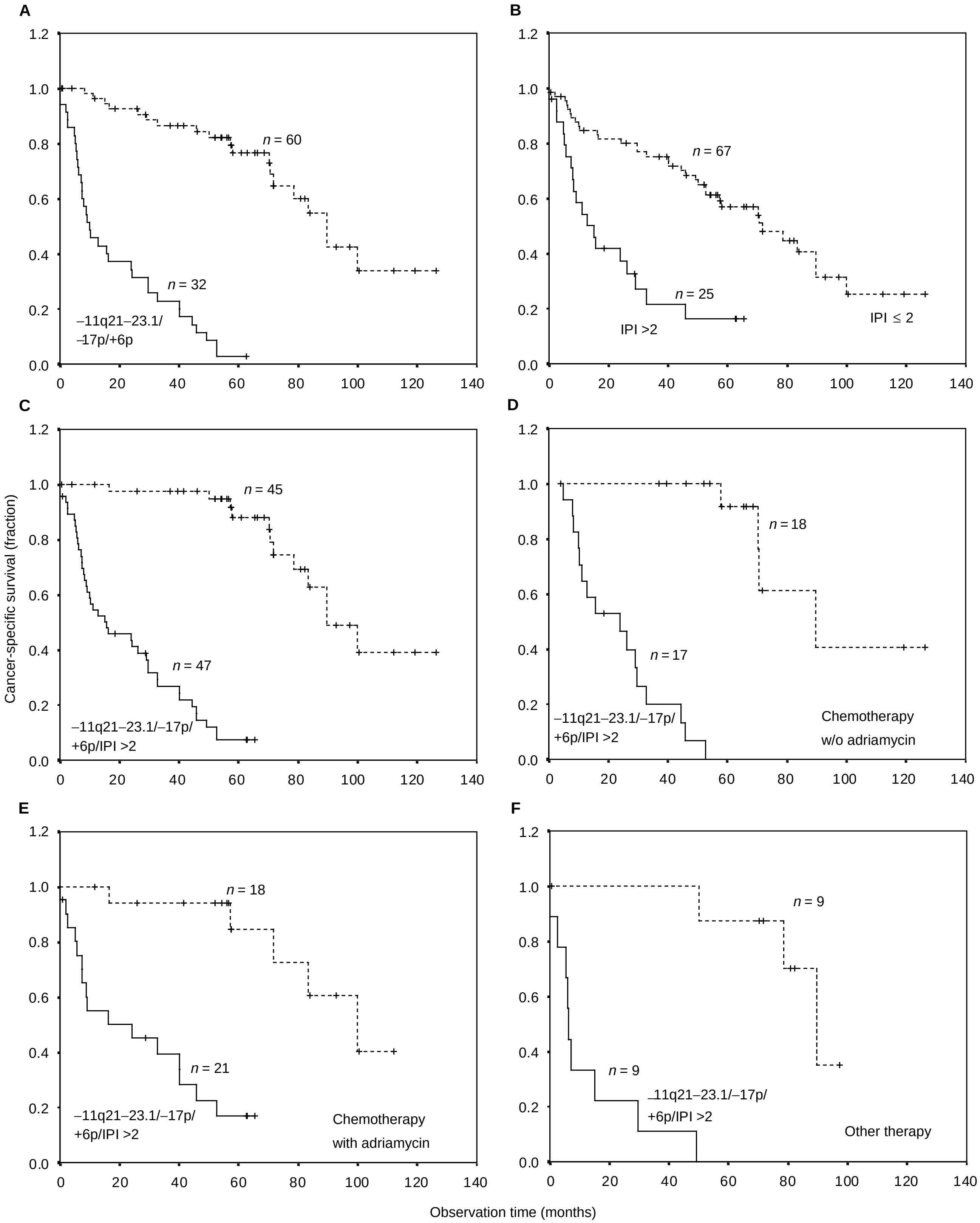

**Figure 5** Survival in NHL according to CGH parameters and IPI status: combination of the parameters and prognostic value for patients receiving different treatments. Kaplan-Meyer plots were generated for all 92 patients for cases with the presence (solid lines), or absence (dotted lines) of –11q21–23.1 and/or –17p and/or +6p (A; relative risk (RR) = 13), IPI > 2 (B; RR = 3.9), and –11q21–23.1 and/or –17p and/or +6p and/or IPI>2 (combined CGH/IPI parameter, C; RR = 26). Survival was also assessed for patients receiving chemotherapy without doxorubicin (D, 35 cases), with doxorubicin (E, 39 cases), or other therapy (F, 18 cases) according to the presence (solid lines), or absence (dotted lines) of –11q21–23.1 and/or –17p and/or +6p and/or IPI > 2 (combined CGH/IPI parameter). *P*-values were less than 0.001 for all comparisons.

 *© 2001 Cancer Research Campaign*

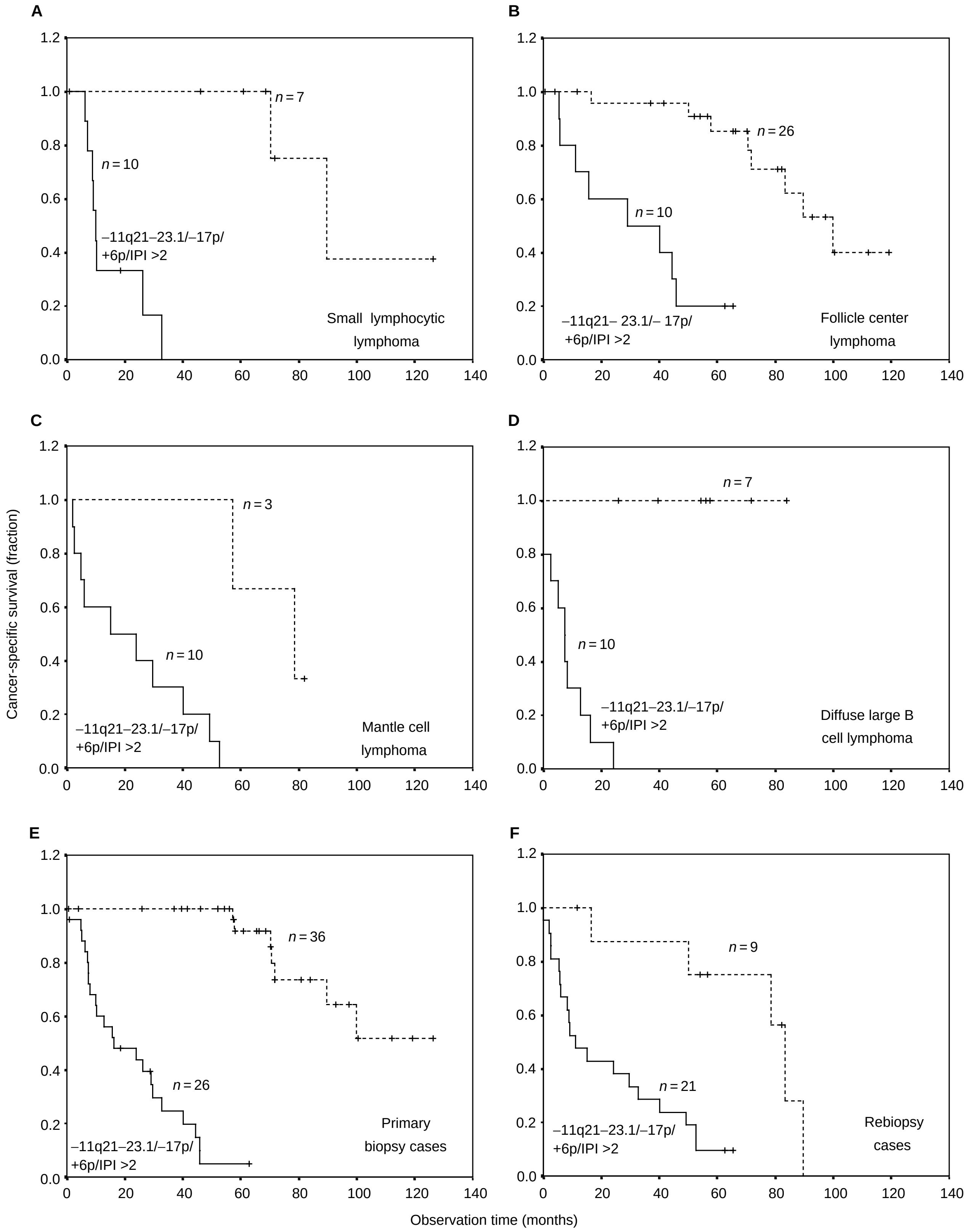

**Figure 6** Survival in NHL according to the combined CGH/IPI parameter: prognostic value for different histologies and primary/recurrent disease. Survival was assessed for patients with small lymphocytic lymphoma (A, 17 cases), follicle centre lymphoma (B, 36 cases), mantle cell lymphoma (C, 13 cases), and diffuse large B-cell lymphoma (D, 17 cases), as well as for the primary biopsy (E, 62 cases) and rebiopsy (F, 30 cases) patients according to the presence (solid lines), or absence (dotted lines) of –11q21–23.1 and/or –17p and/or +6p and/or IPI>2 (combined CGH/IPI parameter). *P*-values were less than 0.001 for all comparisons, except for MCL ($P$ = 0.006).

of the tumour suppressor genes and proto-oncogenes at these loci need yet to be determined.

Another novel finding in our study is that the number of aberrations per tumour showed a bimodal distribution. The tumours with a high number of aberrations (≥ 4) had a high S phase fraction. Also, the patients with tumours with a high number of aberrations had a much shorter survival time than the others, in agreement with the results of classical cytogenetic studies (reviewed by Knutsen, 1998). As expected, most of the aberrations were found more frequently in the tumours with many aberrations. However, several of these presumably secondary aberrations were associated (Table 2), indicating specific oncogene and/or tumor suppressor gene cooperation.

Candidates for primary CGH aberrations in B-cell lymphomas, i.e. aberrations found at a high frequency also in cases with few aberrations, include the gains at 12cen-q15 and 18q13.3–21, and possibly also the 11q21–23.1 deletions in small lymphocytic lymphomas. Among these aberrations, loss of 11q and gain of chromosome 12 have been defined as primary events from classical cytogenetic studies (Mitelman et al, 1994). Interestingly, the gains at 12cen-q15 and 18q13.3–21 did not have any prognostic value, suggesting that additional, 'secondary' events are required for evolution towards a more aggressive malignant lymphoma.

None of the aberrations detected by CGH were specific (i.e. diagnostic) for any lymphoma sub-type. However, several of the aberrations were found at a significantly higher frequency in some lymphoma sub-types, such as +12cen-q15 in SLL, –1p21–31.1, –8p, –9p21-ter, –11q21–23.1, –13q13–21.1 in MCL, and +6p in DLBCL. Gain of chromosome 12 or parts of this chromosome has been observed by others in SLL (Bentz et al, 1995; Autio et al, 1998). However, 12cen–q15 gain was more frequent in SLL in this study (37%). The mantle cell lymphomas typically had a high number of aberrations, and the losses at 1p21–31.1, 8p, 9p21-ter, 11q21–23.1, and 13q13–21.1 were more frequent in this subset of NHL, in concordance with the results of others (Monni et al, 1998; Beà et al, 1999; Bentz et al, 2000). Interestingly, the losses at 1p21–31.1, 8p, 9p21-ter, and 13q13–21.1 typically occurred together (Table 2), and together with cyclin D1 over-expression caused by a t(11;14) translocation (unpublished results). Gain of 3q21-ter was found in almost 50% of the MCL cases, but this aberration is common in all types of NHL, including MZL (Monni et al, 1996; Dierlamm et al, 1997; Monni et al, 1998; Beà et al, 1999; Bentz et al, 2000; Figure 4). Concerning the diffuse large B-cell lymphomas, we found gain of 6p at a similar frequency (24%) as Monni et al (1996; 21%), but a higher incidence of 17p losses (29%) than Monni et al (1996; 3%).

Since patients with B-cell lymphomas with a high S phase fraction have a poor prognosis (Stokke et al, 1998b), it is of particular interest to identify the genetic aberrations responsible for this phenotype. The losses of 8p and 17p, and the gains of 3q21-ter, 6p, 7p and 8q23-ter were associated with a high S phase fraction (Table 4). Four of these aberrations are associated pairwise (–8p, +3q21-ter, +7p, +8q23-ter). Hence, not all four of these aberrations necessarily target genes that are important in the control of cell proliferation. The anti-proliferative activities of wild-type p53 are well documented in the literature (for reviews, see: Sherr, 1996; Levine, 1997), and our previous study showed that a high S phase fraction was characteristic for NHL cases with either mutations in TP53, loss of TP53, or clonal expression of p53 (Stokke et al, 2000).

Several aberrations with prognostic information were found in this study (Table 3). Multivariate analysis showed that loss of 11q21-23.1 (ATM) and 17p (TP53), and gain of 6p by CGH were independent prognostic factors. Other studies have shown that loss of 17p or any aberration in the TP53 pathway are strong prognostic factors in all subtypes of NHL (Sander et al, 1993; Tilly et al, 1994; Dohner et al, 1995; Schoch et al, 1995; Hernandez et al, 1996; Panayiotidis and Kotsi, 1999; Dohner et al, 2000; Stokke et al, 2000; reviewed by Knutsen, 1998), while 11q deletions are unfavorable for patients with SLL/CLL (Dohner et al, 1997; Panayiotidis and Kotsi, 1999; Dohner et al, 2000). Schouten et al (1990) described gain of chromosome 6 as an adverse prognostic sign in a group of patients with all types of NHL.

Interestingly, S phase fraction, presence of BCL1 and BCL2 translocations, lymphoma subtype, biopsy status (primary vs secondary), as well as B symptoms, became non-significant to predict prognosis when entered into Cox multivariate analysis together with the 3 CGH aberrations and IPI (Table 4). An 'and/or' combinatory scheme was found to incorporate the prognostic CGH aberrations and IPI into a single binary parameter which had high prognostic value for patients receiving different treatments, with various lymphoma subtypes, and primary as well as relapse cases (Figures 5, 6). The prognostic chromosomal imbalances identified here clearly warrant large prospective studies (e.g. by FISH) to evaluate whether prognostication and treatment stratification of NHL can be based on chromosomal aberrations.

### ACKNOWLEDGEMENTS

Thanks to Marzieh Beigi for invaluable help with probe preparation and hybridizations. Olav Kaalhus kindly helped with the statistical analysis. This work was supported by The Norwegian Cancer Society.

### REFERENCES

Autio K, Aalto Y, Franssila K, Elonen E, Joensuu H and Knuutila S (1998) Low number of DNA copy number changes in small lymphocytic lymphoma. *Haematologica* **83**: 690–692

Beà S, Ribas M, Hernandez JM, Bosch F, Pinyol M, Hernandez L, Garcia JL, Flores T, Gonzalez M, Lopez-Guillermo A, Piris MA, Cardesa A, Montserrat E, Miro R and Campo E (1999) Increased number of chromosomal imbalances and high-level DNA amplifications in mantle cell lymphoma are associated with blastoid variants. *Blood* **93**: 4365–4374

Bentz M, Huck K, Manoir S, Joos S, Werner CA, Fischer K, Döhner H and Lichter P (1995) Comparative genomic hybridization in chronic B-cell leukemias shows a high incidence of chromosomal gains and losses. *Blood* **85**: 3610–3618

Bentz M, Werner CA, Döhner H, Joos S, Barth TFE, Siebert R, Schroder M, Stilgenbauer S, Fischer K, Moller P and Lichter P (1996) High incidence of chromosomal imbalances and gene amplifications in the classical follicular variant of follicle center lymphoma. *Blood* **88**: 1437–1444

Bentz M, Plesch A, Bullinger L, Stilgenbauer S, Ott G, Muller-Hermelink HK, Baudis M, Barth TF, Moller P, Lichter P and Döhner H (2000) t(11;14)-positive mantle cell lymphomas exhibit complex karyotypes and share similarities with B-cell chronic lymphocytic leukemia. *Genes Chromosomes Cancer* **27**: 285–294

Bullrich F, Rasio D, Kitada S, Starostik P, Kipps T, Keating M, Albitar M, Reed JC and Croce CM (1999) ATM mutations in B-cell chronic lymphocytic leukemia. *Cancer Res* **59**: 24–27

Carbone PP, Kaplan HS, Musshoff K, Smithers DW and Tubiana H (1971) Report of the Committee of Hodgkin's disease staging classification. *Cancer Res* **31**: 1860–1861

DeAngelis P, Clausen OPF, Schjølberg A and Stokke T (1999) Chromosomal gains and losses in primary colorectal carcinomas detected by CGH and their

*© 2001 Cancer Research Campaign*

associations with tumour DNA ploidy, genotypes and phenotypes. *Br J Cancer* **80**: 526–535

Dierlamm J, Rosenberg C, Stul M, Pittaluga S, Wlodarska I, Michaux L, Dehaen M, Verhoef G, Thomas J, de Kelver W, Bakker-Schut T, Cassiman JJ, Raap AK, De Wolf-Peeters C, Van den Berghe H and Hagemeijer A (1997) Characteristic pattern of chromosomal gains and losses in marginal zone B cell lymphoma detected by comparative genomic hybridization. *Leukemia* **11**: 747–758

Dohner H, Fischer K, Bentz M, Hansen K, Benner A, Cabot G, Diehl D, Schlenk R, Coy J and Stilgenbauer S (1995) p53 gene deletion predicts for poor survival and non-response to therapy with purine analogs in chronic B-cell leukemias. *Blood* **85**: 1580–1589

Dohner H, Stilgenbauer S, James MR, Benner A, Weilguni T, Bentz M, Fischer K, Hunstein W and Lichter P (1997) 11q deletions identify a new subset of B-cell chronic lymphocytic leukemia characterized by extensive nodal involvement and inferior prognosis. *Blood* **89**: 2516–2522

Dohner H, Stilgenbauer S, Benner A, Leupolt E, Krober A, Bullinger L, Dohner K, Bentz M and Lichter P (2000) Genomic aberrations and survival in chronic lymphocytic leukemia. *N Engl J Med* **343**: 1910–1916

Fisher RI, Gaynor ER, Dahlberg S, Oken MM, Grogan TM, Mize EM, Glick JH, Coltman CA and Miller TP (1993) Comparison of a standard regimen (CHOP) with three intensive chemotherapy regimens for advanced non-Hodgkin's lymphoma. *N Engl J Med* **328**: 1002–1006

Galteland E, Holte H and Stokke T (1999) c-MYC, RB-1, and TP53 copy number in B-cell Non-Hodgkin's Lymphoma assessed by dual-color fluorescence in situ hybridization. *Cytometry* **38**: 53–60

Harris NL, Jaffe ES, Stein H, Banks PM, Chan JK, Cleary ML, Delsol G, De Wolf Peeters C, Falini B and Gatter KC (1994) A revised European-American classification of lymphoid neoplasms: A proposal from the International Lymphoma Study Group. *Blood* **84**: 1361–1392

Heim S and Mitelman F (1995) *Cancer cytogenetics*. Second Edn. pp 237–309 Wiley-Liss: New York

Hernandez L, Fest T, Cazorla M, Teruya-Feldstein J, Bosch F, Peinado MA, Piris MA, Montserrat E, Cardesa A, Jaffe ES, Campo E and Raffold M (1996) p53 gene mutations and protein overexpression are associated with aggressive variants of mantle cell lymphomas. *Blood* **87**: 3351–3359

Kallioniemi O-P, Kallioniemi A, Piper J, Isola J, Waldman FM, Gray JW and Pinkel D (1994a) Optimizing comparative genomic hybridization for analysis of DNA sequence copy number changes in solid tumors. *Genes Chromosomes Cancer* **10**: 231–243

Kallioniemi A, Kallioniemi O-P, Piper J, Tanner M, Stokke T, Chen L, Smith HS, Pinkel D, Gray JW and Waldman FM (1994b) Detection and mapping of amplified DNA sequences in breast cancer by comparative genomic hybridization. *PNAS* **91**: 2156–2160

Knutsen T (1998) Cytogenetic changes in the progression of lymphoma. *Leuk Lymphoma* **31**: 1–19

Levine AJ (1997) p53, the cellular gatekeeper for growth and division. *Cell* **88**: 323–331

Mitelman F, Kaneko Y and Berger R (1994) Report of the committee on chromosome changes in neoplasia. In: *Human gene mapping 1993*, Cuticchia AJ and Pearson PL (eds) p 773. John Hopkins: Baltimore, MD

Mitelman F, Mertens F and Johansson B (1997) A breakpoint map of recurrent chromosomal rearrangements ain human neoplasia. *Nature Genetics special issue* 417–474

Monni O, Joensuu H, Franssila K and Knuutila S (1996) DNA copy number changes in diffuse large B-cell lymphoma-comparative genomic hybridization study. *Blood* **87**: 5269–5278

Monni O, Joensuu H, Franssila K, Klefstrom J, Alitalo K and Knuutila S (1997) Bcl-2 overexpression associated with chromosomal amplification in diffuse large B-cell lymphoma. *Blood* **90**: 1168–1174

Monni O, Oinonen R, Elonen E, Franssila K, Teerenhovi L, Joensuu H and Knuutila S (1998) Gain of 3q and deletion of 11q22 are frequent aberrations in mantle cell lymphoma. *Genes Chromosomes Cancer* **21**: 298–307

Offit K (1992) Chromosome analysis in the management of patients with Non-Hodgkin's lymphoma. *Leukemia Lymphoma* **7**: 275–282

Ong ST and Le Beau MM (1998) Chromosomal abnormalities and molecular genetics of Non-Hodgkin's lymphoma. *Semin Oncol* **25**: 447–460

Panayiotidis P and Kotsi P (1999) Genetics of small lymphocyte disorders. *Semin Hematol* **36**: 171–177

Pui CH and Evans WE (1998) Acute lymphoblastic leukemia. *N Engl J Med* **339**: 605–615

Rabbitts TH (1994) Chromosomal translocations in human cancer. *Nature* **372**: 143–149

Sander CA, Yano T, Clark HM, Harris C, Longo DL, Jaffe ES and Raffeld M (1993) p53 mutation is associated with progression in follicular lymphomas. *Blood* **82**: 1994–2004

Schaffner C, Idler I, Stilgenbauer S, Döhner H and Lichter P (2000) Mantle cell lymphoma is characterized by inactivation of the ATM gene. *PNAS* **97**: 2773–2778

Schoch C, Rieder H, Stollmann-Gibbels B, Freund M, Tischler HJ, Silling-Engelhardt G and Fonatsch C (1995) 17p anomalies in lymphoid malignancies: diagnostic and prognostic implications. *Leuk Lymphoma* **17**: 271–279

Schouten HC, Sanger WG, Weisenburger DD, Anderson J and Armitage JO (1990) Chromosomal abnormalities in untreated patients with non-Hodgkin's lymphoma: associations with histology, clinical characteristics, and treatment outcome. *Blood* **75**: 1841–1847

Sherr CJ (1996) Cancer cell cycles. *Science* **274**: 1672–1677

Stokke T, Holte H, Smedshammer L, Smeland EB, Kaalhus O and Steen HB (1998a) Proliferation and apoptosis in malignant and normal cells in B-cell Non-Hodgkins lymphomas. *Br J Cancer* **77**: 1832–1838

Stokke T, Smeland EB, Kvaløy S and Holte H (1998b) Tumor cell proliferation, but not apoptosis, predicts survival in B-cell Non-Hodgkins lymphomas. *Br J Cancer* **77**: 1839–1841

Stokke T, Galteland E, Holte H, Smedshammer L, Suo Z, Smeland EB, Børresen-Dale A-L, DeAngelis P and Steen HB (2000) Oncogenic aberrations in the p53 pathway are associated with a high S phase fraction and poor survival in B cell Non-Hodgkin's lymphoma. *Int J Cancer* **89**: 313–324

The International Non-Hodgkin's Lymphoma Prognostic Factors Project (1993) A predictive model for aggressive non-Hodgkin's lymphoma. *N Engl J Med* **329**: 987–994

Tilly H, Rossi A, Stamatoullas A, Lenormand B, Bigorgne C, Kunlin A, Monconduit and Bastard C (1994) Prognostic value of chromosomal abnormalities in follicular lymphoma. *Blood* **84**: 1043–1049

Tirkkonen M, Tanner M, Karhu R, Kallioniemi A, Isola J and Kallioniemi O-P (1998) Molecular cytogenetics of primary breast cancer by CGH. *Genes Chromosom Cancer* **21**: 177–184

*© 2001 Cancer Research Campaign*
